# miR-130a-3p promotes fiber type transition and improves exercise tolerance in mice

**DOI:** 10.1186/s40659-025-00644-z

**Published:** 2025-11-18

**Authors:** Lipeng Xing, Hao Zhou, Haibin Deng, Binghua Yao, Junyi Luo, Ting Chen, Jiajie Sun, Songbo Wang, Gang Shu, Qingyan Jiang, Yongliang Zhang, Qianyun Xi

**Affiliations:** State Key Laboratory of Livestock and Poultry Breeding, Guangdong Provincial Key Laboratory of Animal Nutrition Control, National Engineering Research Center For Breeding Swine Industry, Guangdong Province Research Center of Woody Forage Engineering and Technology, College of Animal Science, South China Agricultural University, No. 483 Wushan Road, Guangzhou, 510642 China

**Keywords:** miR-130a-3p, Skeletal muscle, Exercise tolerance, Oxidative metabolism, TGFβR2

## Abstract

**Background:**

Skeletal muscle plays various roles in physiological stress, such as movement, hormone secretion and oxidative metabolism. MicroRNA (miRNA) is involved in skeletal muscle development and fiber type transformation. Our previous studies have shown that miR-130a-3p is an important regulator of glycolipid metabolism and participates in the aerobic oxidation process. However, its potential impact on skeletal muscle development and muscle fiber type transformation by enhancement of aerobic metabolism of nutrients remains unexplored.

**Methods:**

Mice with knockout and overexpression of miR-130a-3p were utilized to investigate its impact on mouse muscle development via assessments of body composition, metabolic activity in cages, and running performance. The influence of miR-130a-3p on muscle cell proliferation, differentiation, and fiber type was assessed following transfection of C2C12 cells.

**Results:**

In this study, we found that miR-130a-3p overexpression significantly inhibited weight gain and fat content in mice, while promoting oxidative metabolism and improving exercise tolerance. Upregulation of miR-130a-3p in gastrocnemius (GAS) increased the expression of genes controlling cell proliferation, such as proliferating cell nuclear antigen (PCNA), cyclin D, and cyclin E, and decreased the protein expression of myosin heavy chain (MyHC), myogenic differentiation 1 (MyoD) and myopoietin (MyoG). In addition, miR-130a-3p overexpression significantly increased the proportion of type I fibers and decreased the proportion of type IIB fibers in GAS. These phenotype were also observed when miR-130a-3p was overexpressed in differentiated C2C12 myoblasts. Mechanistically, miR-130a-3p can promote the proliferation of myoblasts and inhibit the differentiation of myoblasts. Importantly, miR-130a-3p was proved to target the transforming growth factor beta receptor 2 (TGFβR2). Taken together, miR-130a-3p could be a potential therapeutic target that regulates fiber type conversion during skeletal muscle development and improves skeletal muscle energy metabolism during exercise through regulating TGFβR2 signalling pathway.

## Introduction

MicroRNAs (miRNAs) are a class of non-coding small RNAs and play an important role post-transcriptionally by inhibiting translation and degradation of their target mRNAs [1]. The first miRNA sequence, discovered in Caenorhabditis elegans C, was initially thought to be just a traditional protein-coding gene. However, as research progressed, it was found that this miRNA sequence did not encode a protein, but rather encoded an regulatory RNA with 22 nucleotides [2–4]. More than 2000 miRNAs have been discovered in humans, and according to various research reports, they collectively regulate over one-third of the genes in the human genome [5], and have been shown to participate in metabolic activities such as cell growth, differentiation, development, and apoptosis [6–8]. For instance, miR-125b-5p inhibits the proliferation of 3T3-L1 cells and promotes adipogenic differentiation [9]. MiR-26a can improve peripheral insulin sensitivity and protect β Cell function to alleviate type 2 diabetes [10], and miR-125a-5p can improve the disorder of hepatic glycolipid metabolism in type 2 diabetes by targeting signal transducer and activator of transcription 3 (STAT3) to inhibit hepatic adipogenesis and gluconeogenesis and increase glycogen synthesis [11]. In recent years, there have been reports suggesting that miRNAs are involved in regulating aerobic metabolism processes. Research has shown that miR-181a/b-1 promotes osteogenic differentiation by increasing mitochondrial oxygen consumption rate and ATP-related respiratory metabolism during osteogenesis [12]. MiR-107 promotes lipid accumulation in the liver and reduces glucose tolerance by promoting mitochondrial β-oxidation [13]. Overexpression of miR-146a can promote mitochondria synthesis and respiration in liver cells [14]. MiR-21-5p is involved in regulating mitochondrial respiration and lipid content in H9C2 cells [15], and miR-33a/b inhibits the key enzyme sirtuin 6 (SIRT6), reducing fatty acid oxidation and insulin signaling in the liver [16].

Aerobic metabolism supplies a large amount of energy for the contraction of skeletal muscles. The skeletal muscle, apart from its function in exercise, serves as an important metabolic organ involved in oxidative and energy metabolism. The development and energy utilization of skeletal muscles are influenced by both external environmental factors and internal regulatory factors. Research have shown that moderate aerobic exercise can increase the number of mitochondria and reduce insulin resistance [17]. On the other hand, miRNAs have emerged to play a significant role in the regulation of skeletal muscle development and oxidative metabolism. As early as 2006, research showed that miR-1 can promote myoblast differentiation, while miR-133 stimulates myoblast proliferation [18]. Recent studies have also found that miR-6645p promotes myoblast proliferation and inhibits myoblast differentiation by targeting serum response factors (SRF) and Wnt1 [19], and miR-17-92 promotes proliferation of C2C12 myoblasts but inhibits differentiation [20]. The absence of miR-133a in mouse skeletal muscles can lead to decrease in mitochondrial synthesis and exercise endurance [21] and miR-696 inhibits peroxisome proliferative activated receptor gamma coactivator-1 alpha (PGC-1α) to reduce mitochondrial activity and myoblast oxygen consumption rate in skeletal muscle of mice [22]. The silencing of miR-204-5p in C2C12 myotubes can regulate PGC-1α to enhance mitochondrial biosynthesis [23]. Other studies have found that miR-494-3p can inhibit the formation of rapidly oxidized muscle fibers and reduce the basal oxygen consumption rate of human skeletal muscle by targeting the E1A binding protein P300 (EP300) [24]. and miR-499 changes the type of skeletal muscle fibers in mice and enhances exercise ability and mitochondrial activity, with activation of AMPK/PGC1α signaling in muscle cells by directly targeting folliculin interacting protein 1 (Fnip1) [25].

MiR-130a has long been the subject of significant interest, the existence and function of which were first reported in 2006 [26]. Current researches on miR-130a mainly focus on diseases, immunity and cancer [27]. Studies have shown that exosome miR-130a-3p can regulate the osteogenic differentiation of human adipose stem cells by mediating the SIRT7/Wnt/β-catenin axis [28]. It can also promote chondrocyte proliferation and alleviate osteoarthritis through the PTEN/PI3K/Akt signaling pathway [29], as well as target SKI like proto-oncogene (SnoN) through TGF-β1 pathway to resist renal fibrosis [30]. Bta-miR-130a/b regulates preadipocyte differentiation by targeting peroxisome proliferator activated receptor gamma (PPARγ) and cytochrome P450 family 2 subfamily U member 1 (CYP2U1) in beef cattle [31]. MiR-130a is also proved to promote osteogenic differentiation of human mesenchymal stem cells [32] and reduce liver lipogenesis [33] by targeting PPARγ. In recent years, more reports indicate that miR-130a-3p can directly target TGFBR1 and TGFBR2 in the development of fibrosis in non-alcoholic steatohepatitis, negatively regulating the activation and proliferation of hepatic stellate cells [34]. Studies have also found that miR-130a-3p can inhibit lipid synthesis and metabolism, promote glucose transporter-4 (GLUT4) translocation, and increase cell glucose absorption by targeting PH domain and leucine rich repeat protein phosphatase 2 (PHLPP2) in mice [35], and also target estrogen receptors α (ERα) to inhibit the expression of prolactin (PRL) [36]. These reports indicated the potential involvement of miR-130a in the regulation of energy metabolism. However, the impact of this miRNA on the development and function of skeletal muscle, particularly in relation to aerobic metabolism, has not been reported. Therefore, this study intends to explore the effects of miR-130a-3p on aerobic respiration and exercise ability by employing mice with systemic miR-130a-3p knockout or overexpression, and to provide a preliminary foundation for further research on the regulatory mechanism of miR-130a-3p on skeletal muscle energy metabolism and exercise performance.

## Materials and methods

### Animals

WT mice are specific pathogen free (SPF) grade FVB mice. Systemically miR-130a-3p knockout and overexpressed FVB mice were prepared by Cyagen (Guangzhou) Biotechnology Co., Ltd. using CRISPR/CAS9 gene editing technology [35]. Ten wild-type (WT), miR-130a-3p overexpressed (130OE) and knockout (130KO) mice of 3-week-old and similar growth performance were selected for each group. The mice were housed under a 12 h light/12 h dark cycle at a constant temperature (25 ± 1 °C) with free access to food and water. The animals were fed with high-fat chow (60 kcal% Fat from Research Diets, Cat No. D12492) from 3 to 9 weeks. Weight was recorded once a week. Record the feed intake every 3 days. The body composition and imaging, muscle and exercise endurance of the mice were measured at the end of week 5. The aerobic metabolism of the mice was measured at the end of week 6. After the experiment, the muscle of the mice was collected.

### NMR analysis of the whole-body composition

Body composition was determined on mice using quantitative magnetic resonance (QMR, Niumag Corporation, Shanghai, China). Mice body composition will be assessed following the small animal nuclear magnetic resonance imaging instrument’s guidelines. Prior to testing, mice must have their feces and urine fully voided. Throughout the procedure, mice will be immobilized within the detection cylinder to confine their movement and ensure accurate data acquisition.

### Exercise tolerance test

Before the test, the tilt angle of the treadmill was adjusted to 10 ° with one mouse placed on each track, and then the rest was 10 min after adjusting for 10 min. Each mouse was given an initial speed of 11 m/min and accelerated by 4 m/min every 5 min until the mice sat on the power grid for 5 s, which was judged to be exhausted, and the exercise time and distance of each mouse were recorded [37]. The mouse was allowed to adjust and rest for 20 min before the next test.

### In vivo oxygen consumption assay

O_2_ consumption (VO_2_) and CO_2_ production (VCO_2_) were analyzed utilizing the promotion metabolism measurement system (Sable Systems International, USA). The comprehensive energy metabolism of mice was assessed following the protocols of a small animal metabolism monitoring system. Mice were individually housed in metabolism cages to allow ad libitum access to water and food, while maintaining consistent environmental conditions of temperature, humidity, and circadian rhythm as in the animal facility. A 48-hour acclimatization period in the metabolic cages preceded the 48-hour formal monitoring period during which the mice’s total energy expenditure was measured and recorded before cessation of monitoring.

### Cell lines, culture conditions, transfection

The C2C12 cells were grown in high glucose Dulbecco’s Modified Eagle Medium (DMEM, Gibco) with 10% fetal bovine serum (FBS, Gibco) and 1% penicillin-streptomycin (P/S, Gibco) in a 5% CO_2_ atmosphere at 37 °C. For miR-130a-3p NC/mimics/inhibitor (20 nM) (GenePharma) transfection, C2C12 cells were plated in 12-well dishes at a density of 5 × 10^4^ per well and lipofectamine 2000 (Thermo Fisher) transfection started at the cell density reached 70 to 80%.The sequences of miR-130a-3p NC/mimics/inhibitor were shown in Table 2.

After transfection for 6 h, culture medium (DMEM containing 10% fetal bovine serum and 1% penicillin-streptomycin) was added for cell proliferation, and differentiation culture medium (DMEM containing 2% equine serum and 1% penicillin-streptomycin) was used for differentiation induction for 6 days,. The medium was changed every 2 days.

### CCK8 experiment

The C2C12 cells were inoculated into a 96-well plate at a density of 5 × 10^3^ cells per well. Cells were transfected with miR-130a-3p NC/mimics/inhibitor, and the medium was changed after 6 h. After transfection, the culture continued for 24/48 h. 10 µl CCK-8 (Bioss, Cat No. BA00208) was added to each well, and incubated in an incubator at 37℃ for 1 h. The absorbance at 450 nm of each well was measured with a multifunctional enzyme marker.

### EDU proliferation experiment

With reference to the cell inoculation and transfection methods described above, C2C12 cells were plated in 48-well dishes at a density of 1 × 10^4^ per well. When the cells grew to 80% confluence after transfection, the EDU kit (bioscience, Cat No. C6015XL) was used to detect the cells prolife-ration, and the method was referred to the manufacturer’s instructions.

### Cell/tissue Immunofluorescence

C2C12 cells with good growth condition were selected after 6 d differentiation. First, cells were fixed with 4% paraformaldehyde at room temperature for 1 h. Then cells were treated with 0.4% Triton X-100 for 30 min. The antibody was incubated at 4℃ overnight after 5% goat serum blocking for 1 h. The corresponding fluorescently labeled secondary antibody was added and incubated at room temperature for 1 h in the dark. DAPI was added andincubated at room temperature for 5 min in the dark. The cells were washed with PBS (3 times, 5 min each time) after each step. The photographic statistics were examined by inverted fluorescence microscope. Tissue immunofluorescence refers to the operation steps on cells with additional tissue freezing sections and anti-fluorescence attenuation sealing tablets.

### Dual-luciferase reporter experiments

HEK293T cells were seeded in 96-well plates (Corning) at 2.5 × 10^4^ cells per well and grew overnight to 70%-80% confluence. The dual-luciferase reporter plasmids were co-transfected with miRNA into HEK293T cells and the dosage of miR-130a-3p NC/mimic and wild-type/mutation/deletion dual-luciferase gene reporter vector per well was 3 pmol and 100 ng, respectively. The Dual-Luciferase Reporter Assay System (Promega) was used to detect luciferase activity after 48 h.

### Quantitative real-time PCR

Total RNA was extracted using TRIzol (Thermo Fisher). 1 µg of RNA was converted into complementary deoxyribonucleic acid (cDNA) using a Color Reverse Transcription Kit (EZBioscience, Cat No. A0010CGQ). We performed quantitative real-time PCR (qPCR) using a QuantStudio Real-Time PCR System (Bio-Rad C1000 Touch) using 2 × Real Star Fast SYBR qPCR Mix (GenStar, A301). The mRNA and miRNA internal references were β-actin and U6. The reverse transcription and qPCR primer sequences were shown in Table 1.

### Protein extraction and Western blot analysis

Radioimmunoprecipitation assay (RIPA) buffer containing protease and phosphatase inhibitors (BestBio Cat No. BB-3101) was used to extract proteins. The protein concentration was assessed using the Rapid Gold BCA Protein Assay Kit (Thermo Fisher). Western blotting analysis was performed by loading 15 µg of lysate onto sodium dodecyl sulphate-polyacrylamide gel electrophoresis (SDS-PAGE) gels, transferred the gels to polyvinylidenedifluoride (PVDF) membranes (Millipore), and incubated with rabbit anti-MyHC (1:1000, #MAB4470; R&D Systems), rabbit anti-MyHC Ⅰ (1:1000, #BA-D5; DSHB), rabbit anti-MyHC ⅡB (1:1000, #BF-F3; DSHB), rabbit anti-MyoD (1:1000, #252249; ZEN-BIO), rabbit anti-MyoG (1:1000, #382257; ZEN-BIO) and rabbit anti-Tubulin (1:5000, #bs-20694R; Bioss), goat anti-rabbit secondary antibody (1:50000, # BS13278, Bioworld) conjugated with HRP was used. Tubulin levels served as the loading control. The amount of protein was measured using Image J software.

### Statistical analysis

SPSS 25 and Graphpad prism 9.0 were used for one-way ANOVA and stand-alone sample t-test analysis and plotting. The results were presented as mean ± standard error of the mean (SEM). The significance of difference was judged by a level of *p < 0.05 or **p < 0.01.

## Results

### miR-130a-3p improved exercise tolerance by increasing muscle content

**Fig. 1:**
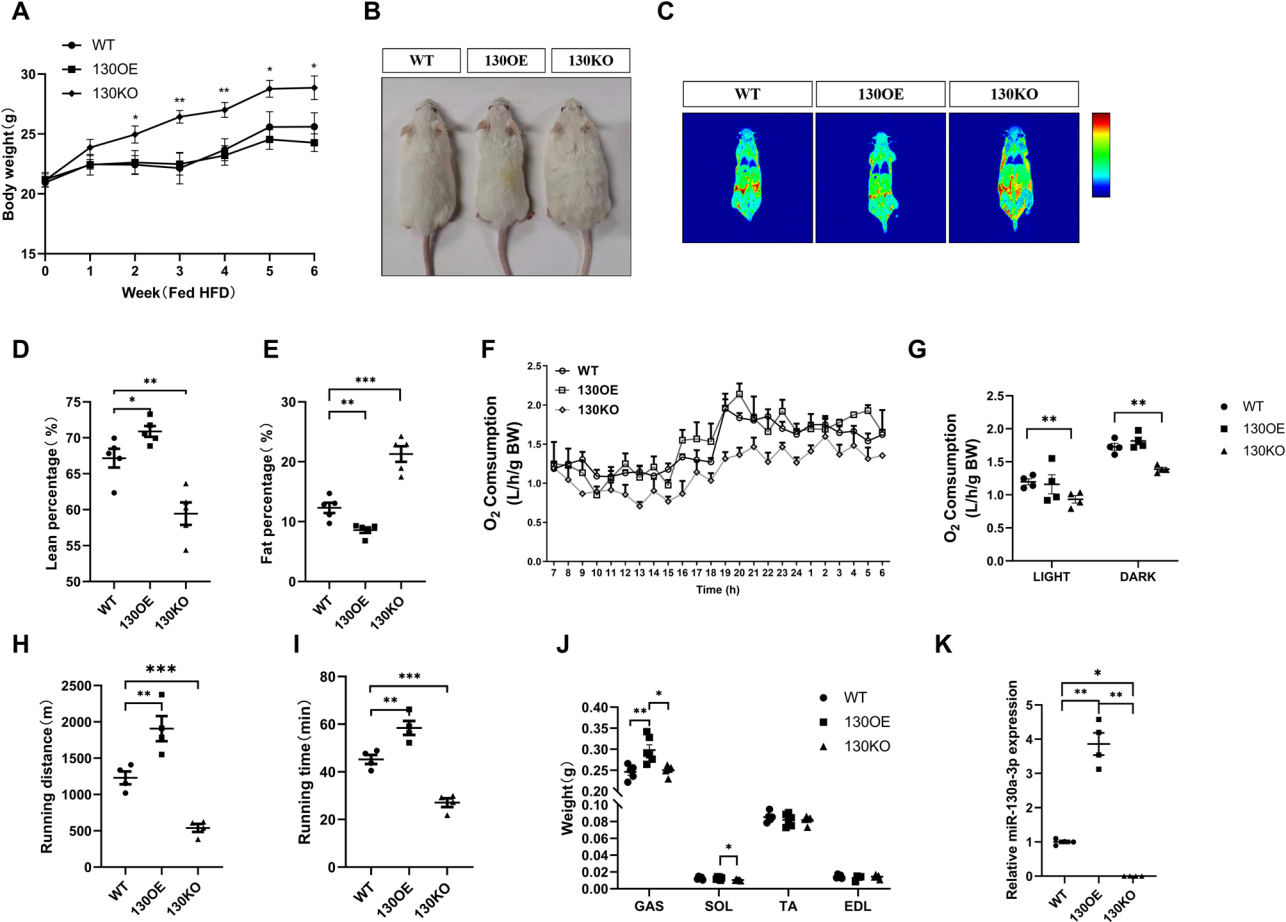
miR-130a-3p improved exercise endurance by increasing muscle content. In the WT, 130OE and 130KO mice, (**A**) The body weight changes of mice in each group under the condition of feeding high fat feed (*n* = 5); (**B**) Mouse body size at week 6 of the experiment; (**C**) Body imaging; (**D**) The lean percentage of body (*n* = 5); (**E**) The fat percentage of body (*n* = 5); (**F-G**) Oxygen consumption (*n* = 4); (**H-I**) exercise capacity (*n* = 5); (**J**) The weight of muscle tissue (*n* = 5); (**K**) The miR-130a-3p expression of GAS (*n* = 5). The data in the figure are all mean ± standard error, with *, * *, and * * * indicating significant differences of *p* < 0.05, *p* < 0.01, and *p* < 0.001 compared to WT group

Starting from the first week of feeding high fat feed, the weight of 130KO mice was significantly greater than that of WT mice, but there is no discernible variance in feed intake across the three groups of mice (Fig. 1A-B). 130OE mice showed an increase in muscle percentage and a decrease in fat percentage, while 130KO mice showed the opposite trend (Fig. 1C-E). The oxygen consumption of three different genotypes of mice was detected. The results showed that the oxygen consumption of 130KO mice was significantly lower than that of 130OE mice (Fig. 1F-G). The above results indicated that knocking out miR-130a-3p can reduce the aerobic respiratory metabolism of mice, and overexpression of miR-130a-3p can improve the aerobic respiratory metabolism of mice, indicating that miR-130a-3p was involved in the body’s aerobic respiratory metabolism activities. The exercise time and distance of 130OE mice were significantly higher than those of WT mice, while the exercise time and distance of 130KO mice were significantly lower than those of WT mice, indicating that overexpression of miR-130a-3p increased the exercise tolerance of mice (Fig. 1H-I). Consistent with the data on body composition, the weight of gastrocnemius muscle in 130OE mice was significantly increased compared to the control group, while the weight of gastrocnemius muscle and soleus muscle in 130KO mice was significantly reduced compared to 130OE mice (Fig. 1J). To confirm that the above changes are caused by changes in miR-130a-3p, we detect miR-130a-3p in mice using qRT-PCR at first. Compared to WT mice, the expression of miR-130a-3p in 130OE mice was significantly increased, while the expression in 130KO mice was significantly decreased (Fig. 1K).

### Increased proliferation and reduced differentiation of myoblasts in the gastrocnemius muscle of 130OE mice

**Fig. 2:**
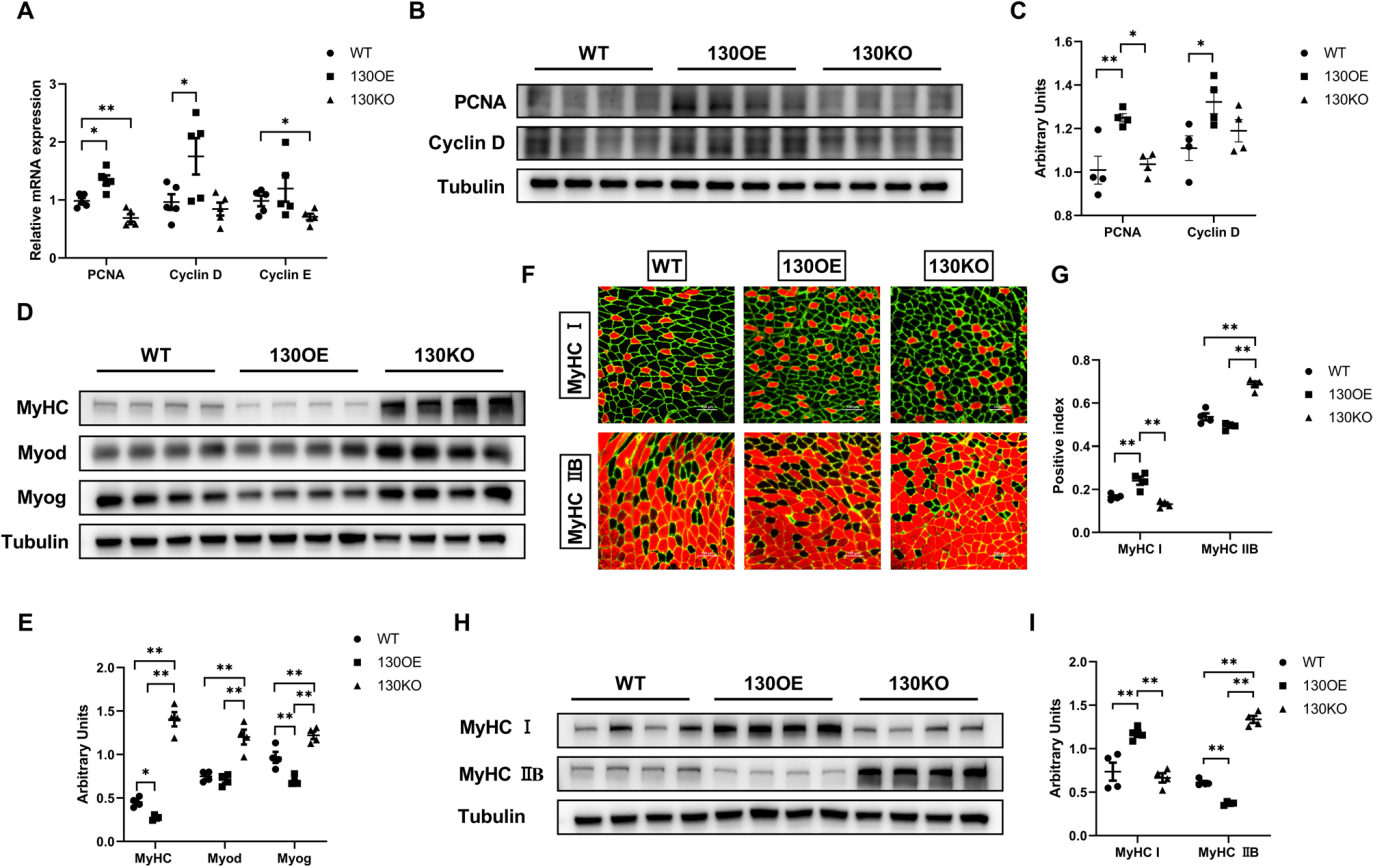
Increased proliferation and reduced differentiation in the gastrocnemius muscle of 130OE mice. In the GAS of WT, 130OE and 130KO mice, (**A-C**) The gene and protein expression of PCNA, Cyclin D and Cyclin E (*n* = 5); (**D-E**) The protein expression of MyHC, MyoD and MyoG (*n* = 4); (**F-G**) Fluorescence diagrams of Type I and IIB fibers; (**H-I**) The protein expression of MyHC I and MyHC IIB (*n* = 4). The data in the figure are all mean ± standard error, with * and * *, indicating significant differences of *p* < 0.05 and *p* < 0.01, compared to WT group

To investigate the effect of miR-130a-3p on skeletal muscle development, we detected the expression of cell proliferation-related genes in mouse gastrocnemius muscle. Compared with WT mice, the expression of PCNA and Cyclin D genes in 130OE mice were significantly increase, while the expression of PCNA and Cyclin E genes in 130KO mice were significantly decrease (Fig. 2A). The results indicated that miR-130a-3p can promote the expression of genes related to skeletal muscle cell proliferation. The protein expression of PCNA and Cyclin D in 130OE mice was significant increased compared with WT mice, while the protein expression of PCNA 130KO mice was also significantly reduced compared with 130OE mice (Fig. 2B-C). To explore whether the beneficial effects of miR-130a-3p in mice was by promoting skeletal muscle differentiation, we detected the expression of gastrocnemius muscle differentiation related proteins in mice. The expression of MyHC/MyoG in 130OE mice were significantly lower than those in WT mice, while the expression levels of MyHC/MyoD/MyoG protein in 130KO mice was significantly higher compared with WT mice and 130OE mice (Fig. 2D-E), indicating that miR-130a-3p inhibited the differentiation of skeletal muscles in mice.

The types of muscle fibers were related to exercise tolerance and aerobic respiratory metabolism. Our results showed that the proportion of type I fibers in 130OE mice was significantly higher than that in WT mice, while it was significantly reduced in 130KO mice. The proportion of type IIB fibers in 130OE mice was significantly lower than that in WT mice, while 130KO mice showed a significant increase (Fig. 2F-G). The expression of MyHC I protein was significantly increased in 130OE mice, while the expression level of MyHC IIB protein was significantly decreased (Fig. 2H-I). The above results indicated that miR-130a-3p can increase the proportion of type I fibers in skeletal muscle while reducing the proportion of type IIB fibers.

### miR-130a-3p promoted proliferation of C2C12 myoblasts

**Fig. 3:**
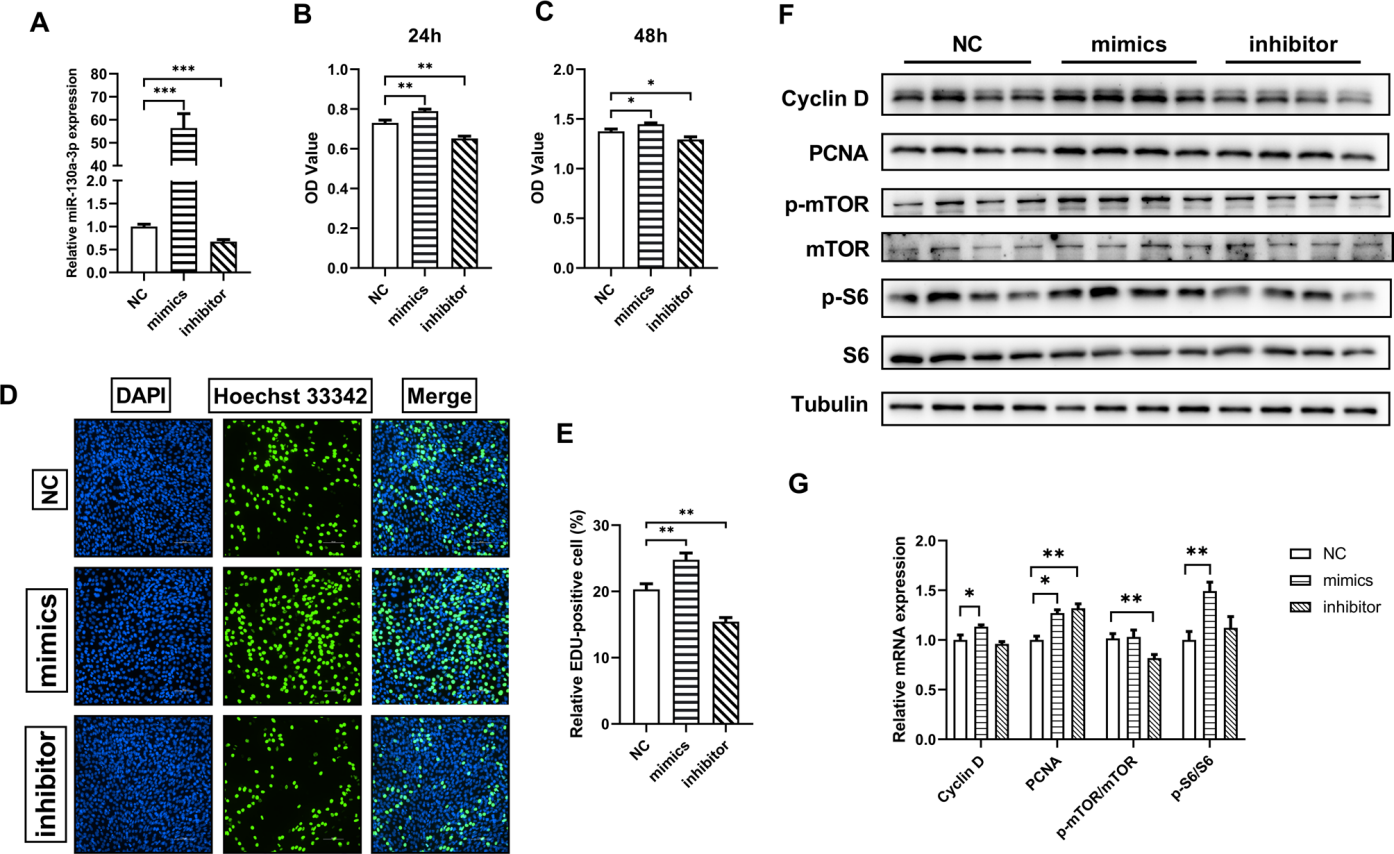
miR-130a-3p promoted proliferation of C2C12 cells. (**A**) The efficiency of C2C12 transfection of miR-130a-3p NC/mimics/inhibitor (*n* = 6); C2C12 cells were transfected with miR-130a-3p NC/mimics/inhibitor and cultured for 24 h (**B**) and 48 h (**C**). The OD values of each group were detected by CCK-8 test (*n* = 6); (**D**) Inoculate C2C12 cells on a 48-well plate, transfect miR-130a-3p NC/mimics/inhibitor, and culture for 24 h. Cell proliferation was detected by EDU method (*n* = 4); (**E**) Fluorescence statistics of cell proliferation detected by EDU method. (**F-G**) Protein expression related to proliferation. The data in the figure are all mean ± standard error, with *, * *, and * * * indicating significant differences of *p* < 0.05, *p* < 0.01, and *p* < 0.001 compared to WT group

In order to further explore the proliferative effects of miR-130a-3p in myoblasts, we conducted CCK-8 and EDU tests in C2C12 cells. C2C12 cells were transfected with miR-130a-3p NC/mimics/inhibitor, and CCK-8 assay was performed after culture for 24–48 h. Before the start of CCK-8 and EDU experiments, C2C12 cells were cultured to measure transfection efficiency. The expression of miR-130a-3p significantly increased in the transfected mimics group, while it decreased significantly in the inhibitor group (Fig. 3A). It can be seen that the OD value of the mimics group was significantly higher than that of the NC group and the inhibitor group was significantly lower than NC group after 24 h of cultivation after transfection (Fig. 3B). The results that cultured for 48 h after C2C12 transfection were consistent with the above results. (Fig. 3C). The EDU test results were shown in Fig. 3D&E, where the proportion of EDU positive cells in the mimics group was significantly higher than that in the NC group, while the inhibitor group was significantly lower than that in the NC group. The above results indicated that overexpression of miR-130a-3p can promote the proliferation of C2C12 cells.

### miR-130a-3p decreased the fusion of differentiated C2C12 myoblasts

**Fig. 4:**
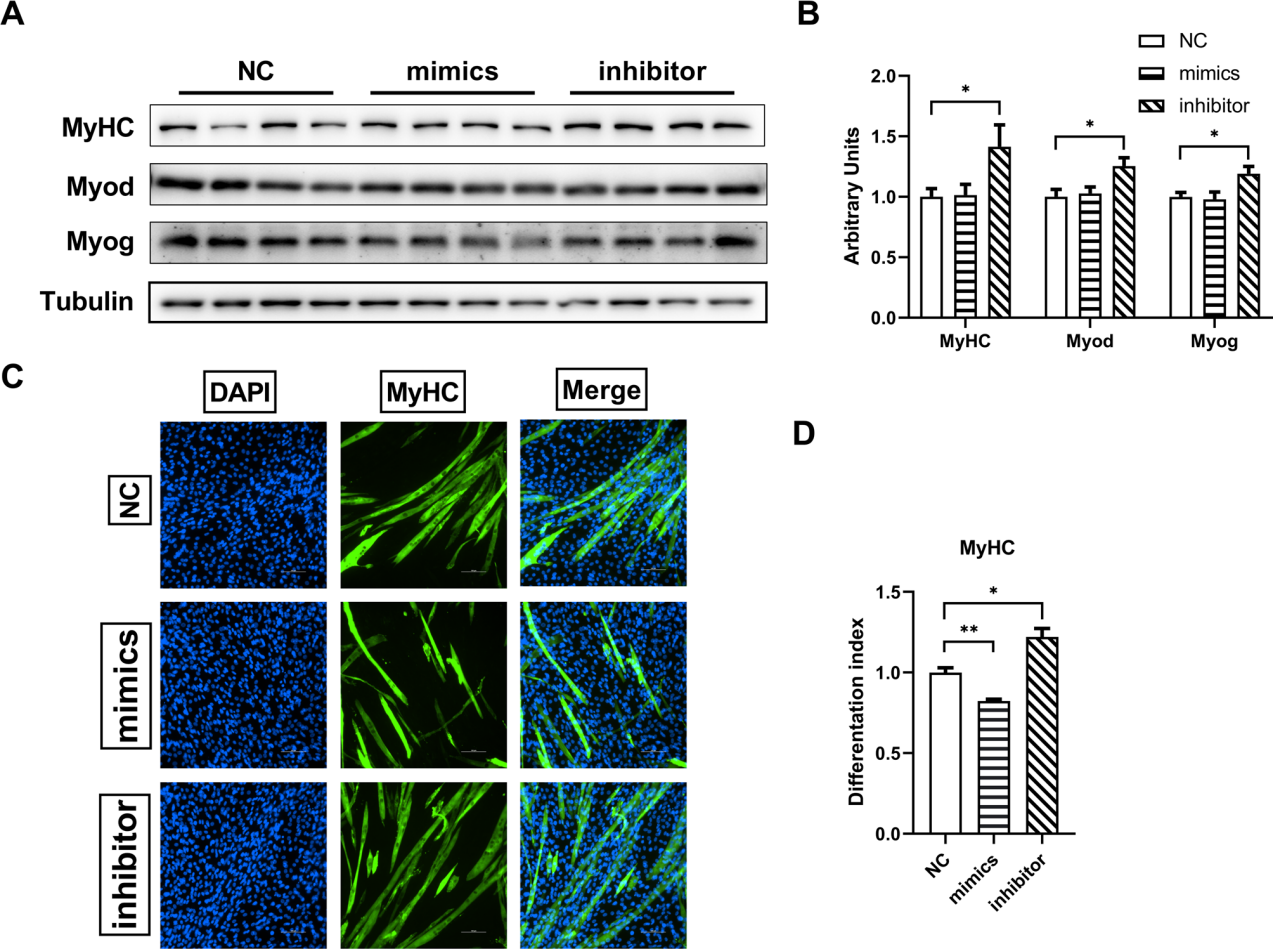
miR-130a-3p decreased the fusion of differentiated C2C12 cells. C2C12 cells were transfected with miR-130a-3p NC/mimics/inhibitor 6 days after differentiation, (**A**) Western blot bands of differentiation-related proteins MyHC/MyoD/MyoG were obtained (*n* = 8); (**B**) Western blot strip statistics of MyHC/MyoD/MyoG protein; (**C**) Immunofluorescence maps of C2C12 cells (*n* = 4); (**D**) Immunofluorescence statistics of MyHC protein. The data in the figure are all mean ± standard error, with * and * *, indicating significant differences of *p* < 0.05 and *p* < 0.01, compared to WT group

C2C12 cells were inoculated with 12-well plates, detecting the muscle differentiation-related proteins after transfected with miR-130a-3p NC/mimics/inhibitor respectively and differentiated for 6 days. As shown in Fig. 4A&B, MyHC protein was significantly increased in inhibitor group, and differentiation marker proteins MyoD and MyoG were also significantly increased, but there was no significant difference between the mimics group and the NC group. Subsequently, the effect of miR-130a-3p on C2C12 cell differentiation was further verified by immunofluorescence in this study. In the mimics group, the myoduct integration was significantly reduced, while the myoduct integration was significantly increased in the inhibitor group (Fig. 4C-D). The evidence showed that miR-130a-3p decreased the fusion of differentiated C2C12 cells.

### miR-130a-3p increased the proportion of type I fibers with strong oxidation capacity

**Fig. 5:**
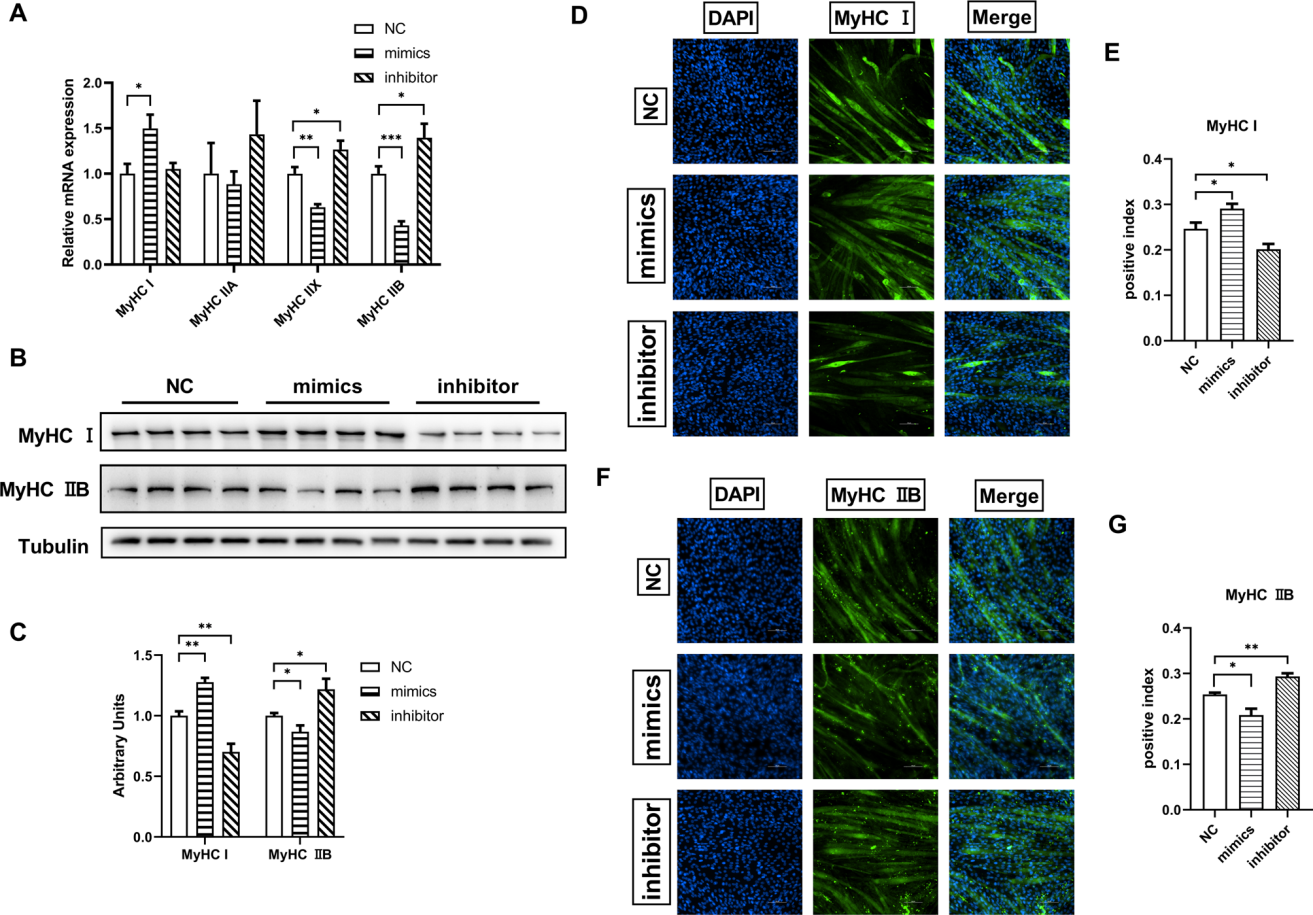
miR-130a-3p increased the proportion of type I fibers with strong oxidation capacity. C2C12 cells after transfection with miR-130a-3p NC/mimics/inhibitor at 6 days after differentiation (**A**) Expression of muscle fiber types (*n* = 6); (**B**) Western blot bands of type I and type IIB fibers (*n* = 6); (**C**) Western blot strip statistics of MyHC I and MyHC IIB proteins; (**D**) Immunofluorescence of type I fibers (*n* = 4); (**E**) Immunofluorescence statistics of type I fibers; (**F**) Immuno-fluorescence map of IIB fibers (*n* = 4). (**G**) Immunofluorescence statistics of type IIB fibers. The data in the figure are all mean ± standard error, with *, * *, and * * * indicating significant differences of *p* < 0.05, *p* < 0.01, and *P* < 0.001 compared to WT group

To further explore the effect of miR-130a-3p on the differentiation process of C2C12 cells, the gene expression of different muscle fiber types and the proportion of type I and type IIB fibers of differentiated C2C12 cells were detected in this experiment. Transfection of miR-130a-3p mimics could significantly increase the expression of MyHC I gene (oxidized fiber), while significantly decrease the expression of MyHC IIX/MyHC IIB gene (glycolytic fiber). However, MyHC IIX/MyHC IIB gene expression were significantly increased in the transfection inhibitor group (Fig. 5A). miR-130a-3p mimics significantly increased the protein expression of MyHC I, but inhibited the protein expression of MyHC IIB. However, MyHC I protein expression was significantly inhibited and MyHC IIB protein expression was significantly increased with miR-130a-3p inhibitor (Fig. 5B-C). miR-130a-3p mimics significantly increased the positive proportion of MYHC I fiber and significantly decreased the positive proportion of MyHC IIB fiber, and showed the opposite for miR-130a-3p inhibitor group (Fig. 5D-G). The results showed that miR-130a-3p could increase the proportion of type I fibers with strong oxidation capacity and decrease the proportion of type IIB fibers with weak oxidation capacity.

### miR-130a-3p promoted exercise tolerance by targeting TGFβR2

**Fig. 6:**
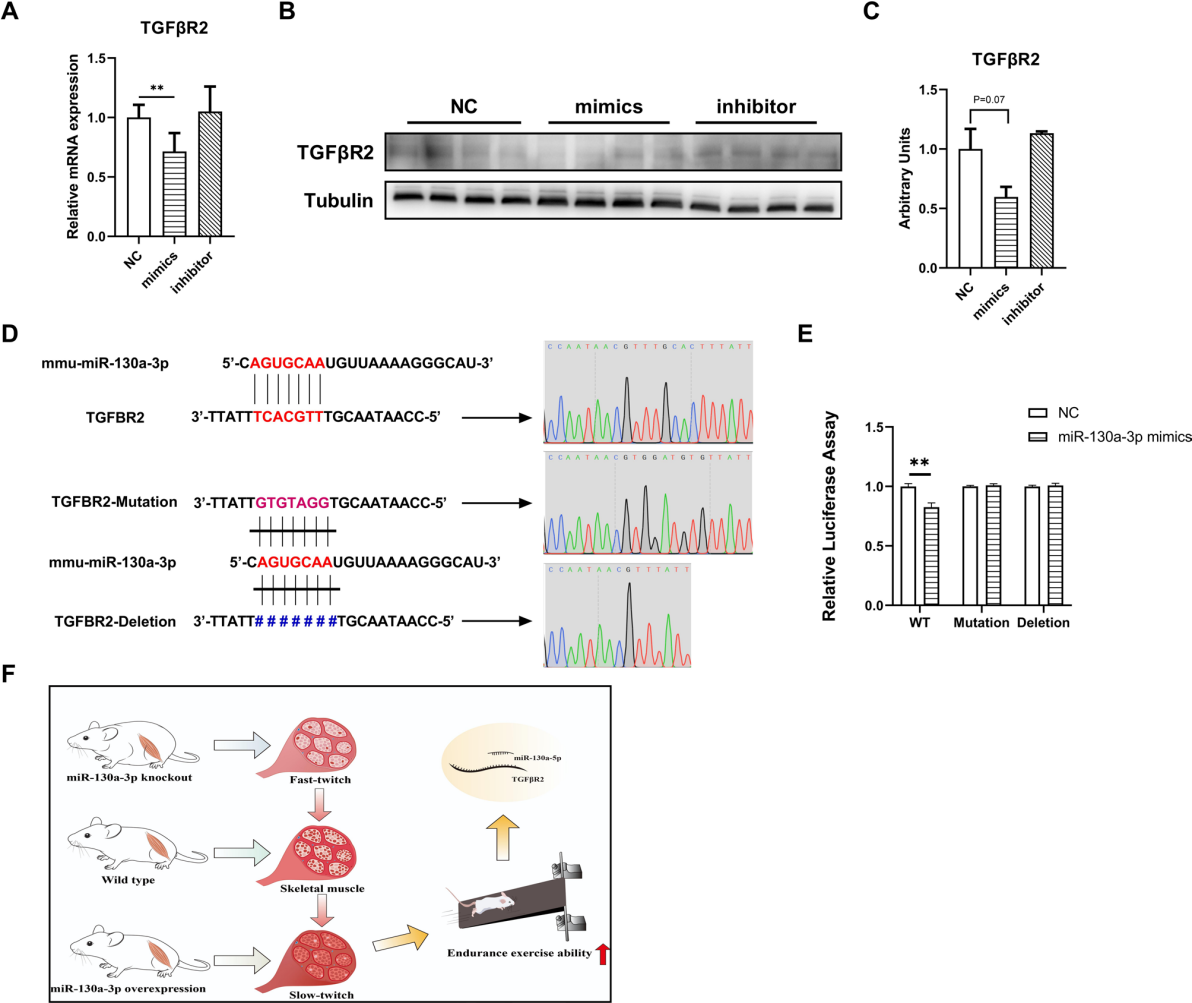
miR-130a-3p promoted exercise tolerance by targeting TGFβR2. C2C12 cells after transfection with miR-130a-3p NC/mimics/inhibitor at 6 days after differentiation, (**A**) The gene expression of TGFβR2 (*n* = 6); (**B**) Western blot bands of TGFβR2 (*n* = 4); (**C**) Western blot strip statistics of TGFβR2; (**D**) miR-130a-3p had a target interaction with the 3’UTR of TGFβR2; (**E**) Statistical chart of miR-130a-3p and TGFβR2 dual luciferase validation fluorescence values (*n* = 8); (**F**) Mechanism diagram of miR-130a-3p regulating fiber type transformation to increase exercise endurance in mice. The data in the figure are all mean ± standard error, with * and * *, indicating significant differences of *p* < 0.05 and *p* < 0.01, compared to WT group

miRNA played a regulatory role by binding to the 3 ‘UTR region of the target gene. To explore the molecular mechanism how miR-130a-3p regulated muscle fiber types and increased exercise tolerance, we predicted the target genes of miR-130a-3p on the website (https://mirdb.org/mirdb/index.html) and focused on the TGFβR2 gene. Results showed that overexpression of miR-130a-3p could significantly reduce the gene expression of TGFβR2 (Fig. 6A), and the protein expression of TGFβR2 had a reduced trend (*P* = 0.07, Fig. 6B-C). According to the binding region of miR-130a-3p and TGFβR2, dual luciferase plasmids containing binding region (WT), mutation-binding region (Mut) and deletion binding region (Del) were designed and constructed (Fig. 6D). Experimental results showed that luciferase activity was significantly reduced when miR-130a-3p mimics was co-transfected with WT plasmid, and the luciferase activity of the other groups d not change significantly (Fig. 6E). The above results demonstrated that miR-130a-3p directly targeted TGFβR2, and affect muscle fiber types possibly by regulating TGFβR2 signaling to increase exercise tolerance (Fig. 6F).

**Table 1:** RNA reverse transcription and quantitative primer sequence

RNA	Sequences (5’ to 3’)
OligodT18	TTTTTTTTTTTTTTTTTT
miR-130a-3p	CTCAACTGGTGTCGTGGAGTCGGCAATTCAGTTGAGATGCCCTT
qPCR	Sequences(5’ to 3’)
miR-130a-3p	F: ACACTCCAGCTGGGCAGTGCAATGTTAAAA
	R: TGGTGTCGTGGAGTCG
PCNA	F: TTTGAGGCACGCCTGATCC
	R: GGAGACGTGAGACGAGTCCAT
Cyclin D	F: CTCCGTATCTTACTTCAAGTGCG
	R: CTTCTCGGCAGTCAAGGGAA
Cyclin E	F: GAGCTTGAATACCCCAGGACTG
	R: CGTCTCTCTGTGGAGCTTATAGAC
MyHC I	F: TCCTGCGGAAGTCTGAGAAG
	R: GACACGATCTTTGGCCTTGAC
MyHC IIA	F: CCAGAAGCCTAAGGTGGTCA
	R: AGTTTGTGCCCCAGAGAAGA
MyHC IIX	F: GCGAATCGAGGCTCAGAACAA
	R: GTAGTTCCGCCTTCGGTCTTG
MyHC IIB	F: ATCCGGGTTGAAGACTCTGG
	R: GTGGGTGCTCTTCAAGTTGG
GAPDH	F: AGGTCGGTGTGAACGGATTTG
	R: TGTAGACCATGTAGTTGAGGTCA

**Table 2:** SiRNA sequences for C2C12 cells transfection

	Sequences(5’ to 3’)
NC	sense: UUCUCCGAACGUGUCACGUTT
	antisense: ACGUGACACGUUCGGAGAATT
miR-130a-3p mimics	sense: CAGUGCAAUGUUAAAAGGGCAU
	antisense: GCCCUUUUAACAUUGCACUGUU
miR-130a-3p inhibitor	AUGCCCUUUUAACAUUGCACUG

## Discussion

Skeletal muscle growth and development represent intricate and tightly regulated physiological processes. As the quantity of individual myoblasts rises, their proximity to neighboring myoblasts diminishes, leading to fusion and the creation of binuclear myotubes. Subsequently, these myotubes merge with adjacent myoblasts or myotubes, culminating in the formation of multinuclear myotubes [38, 39]. TGF-beta signaling inhibits cell fusion independently of cellular motility and intercellular contact formation. Conversely, blocking TGFβ signaling promotes cell fusion between mouse and human myotubes and facilitates myotube branching. In vivo administration of exogenous TGFβ protein impairs muscle function and suppresses the formation of enlarged muscle fibers mediated by TGFβR2 during muscle regeneration [40]. Our study identified TGFβR2 as a target gene of miR-130a-3p. Overexpression of miR-130a-3p suppresses TGFβR2 expression, leading to the inhibition of TGFβ signaling. This mechanism potentially underlies the involvement of miR-130a-3p in muscle fiber type transition mediated by TGFβ signaling. Muscle contraction, irrespective of the exercise modality, has been demonstrated to regulate cellular transcription and translation through various mechanisms at both genomic and post-genomic levels, underpinning the adaptive response to exercise [41]. Muscle-specific miRNAs such as miR-1, miR-133a/b, miR-206, and miR-499 are modulated by essential amino acid consumption, endurance exercise, and endurance exercise training. The expression of these miRNAs is linked to anabolic intracellular signaling and muscle hypertrophic responses related to resistance exercise training [42]. Following four weeks of intense treadmill running in mice, circulating levels of miR-133 rose, while levels of miR-133 in muscle tissue decreased. This phenomenon could play a crucial role in the adaptation to exercise-induced motion. Moreover, secreted miRNAs might also have paracrine or endocrine effects on various tissues and organs [43]. In summary, exercise affects miRNA expression, and specific miRNAs regulate gene expression to complete exercise adaptation.

Skeletal muscle consists of thousands of muscle cells with contractile functions, known as muscle fibers [44]. It obtains energy for contraction through both anaerobic and aerobic metabolic pathways. The latter generates much more energy compared to the former, rendering it the most efficacious source of ATP for skeletal muscle [45]. As the main fuels for skeletal muscle oxidation during long-term exercise, a large extent on the intensity and duration of exercise depends to the source of carbohydrate. With the increase of exercise intensity, the contribution of carbohydrate is greater, and improving the usage rate of carbohydrate during long-term exercise through intake of carbohydrate has always been the leading field of sports nutrition research [46]. It has been reported that oxidative muscle fibers have higher glucose sensitivity and GLUT4 protein expression than glycolytic muscle fibers, taking up more glucose under basal and insulin stimulation conditions [47]. Previous studies have shown that miR-130a-3p can promote the translocation of GLUT4 and increase glucose uptake by cells [35]. Our results showed that overexpression of miR-130a-3p in mice improved exercise tolerance and increased muscle content, which agrees with previous reports, and also suggested that this miRNA might be involved in muscle fiber conversion.

Under normal conditions, the number of muscle fibers in skeletal muscle is already established before birth, and the growth and development of muscle after birth mainly reflects in the transformation of muscle fiber types and changes in size, which may transpire throughout the whole process of growth and development of the body and even after maturity [48, 49]. The classification methods of muscle fiber types are constantly evolving with the advancements in biology. Variations in mitochondrial content, ATPase activity and aerobic metabolic enzyme activity are observed in different muscle fiber types [50]. At present, muscle fibers can be categorized into oxidative or glycolytic fibers according to their metabolic type and enzyme activity content. Oxidative muscle fiber has more capillaries and myoglobin, resuling in a redder appearance, commonly referred to as red muscle. It contains more cytochromes, oxidative metabolic enzymes and mitochondria, as well as a slower contraction rate [51, 52]. Conversely, glycolytic muscle fiber, also known as white muscle, exhibit lower levels of myoglobin and capillaries, as well as reduced cytochromes, oxidative metabolic enzymes and mitochondria and contraction rate [53]. Due to these disparities, oxidative muscle fibers show stronger anti-fatigue ability than glycolytic muscle fibers. Previous studies have shown that skeletal muscle fibers affect the endurance level of the body, and the skeletal muscle of elite long-distance runners contains a higher proportion of oxidative muscle fibers [54]. Additionally, the size, quantity and composition of muscle fibers, especially the ratio of oxidative and glycolytic fibers, are associated with physiological aging and pathological muscle atrophy [55]. It has been reported that patients with muscular dystrophy possess lower contents of oxidative fibers, accompanied by a phenomenon of fiber conversion from oxidative to glycolytic [56]. These findings suggest that the proportion of muscle fiber types is strongly associated with muscle diseases. Therefore, increasing the proportion of oxidizing fiber is of great significance for the prevention and control of muscle diseases.

In recent years, studies have shown that long-term endurance exercise can enhance enzyme activities associated with aerobic oxidative metabolism, increase mitochondrial content and promote the aerobic oxidative metabolism of muscle fibers [57]. It has also been reported that a high-fat diet can increase the mitochondrial content in skeletal muscle of mice, significantly improving the exercise endurance performance during running [58]. With the in-depth study of miRNAs, various miRNAs are reported to be involved in the transformation between muscle fiber types. MiR-152 promotes the formation of slow muscle fibers by targeting the uncoupling protein 3 (UCP3) [59]. MiR-151-3p regulates slow muscle gene expression by targeting a slow skeletal and cardiac muscle specific Ca(2+) ATPase (ATP2a2) in skeletal muscle cells [60]. miR-22-3p regulates the conversion of skeletal muscle fiber types by inhibiting the AMPK/SIRT1/PGC-1α signaling pathway [61]. The results of this study showed that miR-130a-3p overexpressed mice have higher exercise tolerance and stronger aerobic metabolism, and verified that miR-130a-3p regulate muscle fiber type conversion and increase the proportion of oxidative fibers. We further demonstrated the regulatory relationship between miR-130a-3p and TGFβR2, which suggested this miRNA is involved in enhancing exercise endurance in skeletal muscle of mice by regulating TGFβR2 signalling pathway.

## Conclusion

In conclusion, our results suggested the important role of miR-130a-3p in the transformation of muscle fiber types. A high level of miR-130a-3p is helpful to increase the ratio of type I fibers and improve exercise tolerance, which is expect to be a potential therapeutic target that regulates fiber type conversion during skeletal muscle development and improves skeletal muscle energy metabolism during exercise.

## Data Availability

The datasets used and/or analyzed during the current study are available from the corresponding author on reasonable request.
